# TBK1 is involved in programmed cell death and ALS-related pathways in novel zebrafish models

**DOI:** 10.1038/s41420-025-02374-3

**Published:** 2025-03-12

**Authors:** Quentin Raas, Gregoire Haouy, Hortense de Calbiac, Elena Pasho, Anca Marian, Ida Chiara Guerrera, Marion Rosello, Patrick Oeckl, Filippo Del Bene, Alberto Catanese, Sorana Ciura, Edor Kabashi

**Affiliations:** 1 Laboratory of Translational Research for Neurological Disorders, Imagine Institute, INSERM UMR 1163, Université Paris Cité, 75015 Paris, France; 2 Proteomics Platform 3P5-Necker, - Structure Fédérative de Recherche Necker, Inserm US24/CNRS UAR 3633, Université Paris Cité, 75015 Paris, France; 3 Sorbonne Université, INSERM U968, CNRS UMR 7210, Institut de la Vision, Paris, France; 4 German Center for Neurodegenerative Diseases (DZNE) Ulm, Ulm, Germany; 5 Department of Neurology, Ulm University Hospital, Ulm, Germany; 6 Institute of Anatomy and Cell Biology, Ulm University School of Medicine, Ulm, Germany

**Keywords:** Genetics of the nervous system, Amyotrophic lateral sclerosis, Experimental models of disease, Cell death in the nervous system

## Abstract

Pathogenic mutations within the TBK1 gene leading to haploinsufficiency are causative of amyotrophic lateral sclerosis (ALS). This gene is linked to autophagy and inflammation, two cellular mechanisms reported to be dysregulated in ALS patients, although its functional role in the pathogenesis could involve other players. We targeted the TBK1 ortholog in zebrafish, an optimal vertebrate model for investigating genetic defects in neurological disorders. We generated zebrafish models with invalidating tbk1 mutations using CRISPR-Cas9 or tbk1 knockdown models using antisense morpholino oligonucleotide (AMO). The early motor phenotype of zebrafish injected with tbk1 AMO beginning at 2 days post fertilization (dpf) is associated with the degeneration of motor neurons. In parallel, CRISPR-induced tbk1 mutants exhibit impaired motor function beginning at 5 dpf and increased lethality beginning at 9 dpf. A metabolomic analysis showed an association between tbk1 loss and severe dysregulation of nicotinamide metabolism, and incubation with nicotinamide riboside rescued the motor behavior of tbk1 mutant zebrafish. Furthermore, a proteomic analysis revealed increased levels of inflammatory markers and dysregulation of programmed cell death pathways. Necroptosis appeared to be strongly activated in TBK1 fish, and larvae treated with the necroptosis inhibitor necrosulfonamide exhibited improved survival. Finally, a combined analysis of mutant zebrafish and TBK1-mutant human motor neurons revealed dysregulation of the KEGG pathway “ALS”, with disrupted nuclear-cytoplasmic transport and increased expression of STAT1. These findings point toward a major role for necroptosis in the degenerative features and premature lethality observed in tbk1 mutant zebrafish. Overall, the novel tbk1-deficient zebrafish models offer a great opportunity to better understand the cascade of events leading from the loss of tbk1 expression to the onset of motor deficits, with involvement of a metabolic defect and increased cell death, and for the development of novel therapeutic avenues for ALS and related neuromuscular diseases.

## Introduction

TANK-binding kinase 1 (TBK1) is a serine/threonine kinase that is constitutively expressed and involved in various cellular processes, including innate immunity and autophagy. Upon stimulation of pathogen recognition receptors, TBK1 participates in the antiviral response and stimulates the production of type-I IFN [1]. TBK1 is a known regulator of autophagy that phosphorylates and controls the activity of the cargo proteins p62, OPTN, NDP52 and interacts with the ULK1 initiation complex [2, 3]. More recently, TBK1 has been recognized as a central regulator of necroptosis; TBK1 phosphorylates RIPK1 and prevents TNF-induced cell death [4]. Interestingly, *TBK1* homozygous loss of function mutations were reported in four patients and was associated with sufficient IFN-I induction but TNF-driven autoinflammation is observed and translates into increased rates of necroptosis in patient-derived fibroblasts [5].

Amyotrophic lateral sclerosis (ALS) is a fatal neurodegenerative disease characterized by gradual loss of motor neurons in the cerebral cortex, brainstem, and spinal cord leading to progressive paralysis with a devastatingly rapid course. ALS shares clinical, genetic, and pathological similarities with frontotemporal dementia (FTD), and approximately 10% of patients affected by ALS have genetic risk factors. Whole-exome sequencing in familial ALS and FTD patients revealed significant enrichment of monoallelic loss-of-function mutations in the gene encoding TBK1 [6]. *TBK1* mutations are estimated to explain or contribute to between 1 and 1.8% of ALS cases and up to 4% of familial ALS/FTD cases, outlining a causal role of TBK1 haploinsufficiency in ALS [6–9].

In the context of ALS, autophagy, innate immunity and programmed cell death have all been extensively studied and shown to be key mechanisms in pathogenesis [10, 11]. Human motor neurons derived from induced pluripotent stem cells (iPSCs) with TBK1 mutations exhibit reduced viability and accumulation of p62-positive cytosolic inclusions [12]. Furthermore, homozygous *Tbk1* inactivation in mice causes death at embryonic day 14.5 due to massive liver degeneration and apoptosis, emphasizing the important role of TBK1 during development [13]. To overcome this lethality, mouse models featuring conditional deletions of Tbk1 have been developed [14]. Interestingly, selective depletion of *Tbk1* in motor neurons did not cause neurodegeneration, while loss of TBK1 activity in other cell types, particularly immune cells, affected the physiopathology [15–17]. Heterozygous Tbk1 deletion alone does not lead to signs of motor neuron degeneration in mice. Surprisingly, this study also describes a subtle role of TBK1 in the modulation of brain inflammation and stresses a bivalent role of TBK1 in SOD1^G93A^ ALS mice [18]. In *Tbk1*^-/−^ mice, TBK1 was shown to be an endogenous inhibitor of RIPK1 which mediates the embryonic lethality [19].

We aimed at investigating the numerous unexplored pathways affected by TBK1 loss of function that could be critically involved in ALS. To overcome the lack of in vivo models and offer a valid alternative for explorative approaches, we developed zebrafish genetic models featuring tbk1 deficiency through two complementary approaches, either the use of antisense morpholino oligonucleotide (AMO) or CRISPR-Cas9, to inactivate the ortholog of this gene. Zebrafish models have been successfully generated to study genetic dysregulation associated with the development of ALS [20, 21]. The single TBK1 ortholog in zebrafish (*Danio rerio*), tbk1, shares 71.39% amino acid sequence identity with the human sequence, with very high conservation of functional domains [22]. Notably, more than 95% sequence identity was observed between the kinase domain of zebrafish and that of human TBK1 and oligomeric structure prediction are suggestive of a functional dimeric form (Sup. Fig. 1), suggesting the conservation of tbk1 biology in zebrafish.

Here, we combined functional and multiomic analysis of tbk1-deficient zebrafish, which revealed important previously unexplored disease mechanisms and that contribute to the understanding of how TBK1 mutations affect vulnerable neurons in ALS.

## Results

### Morpholino-induced *tbk1* knockdown induces an early phenotype with decreased motor function

**Fig. 1:**
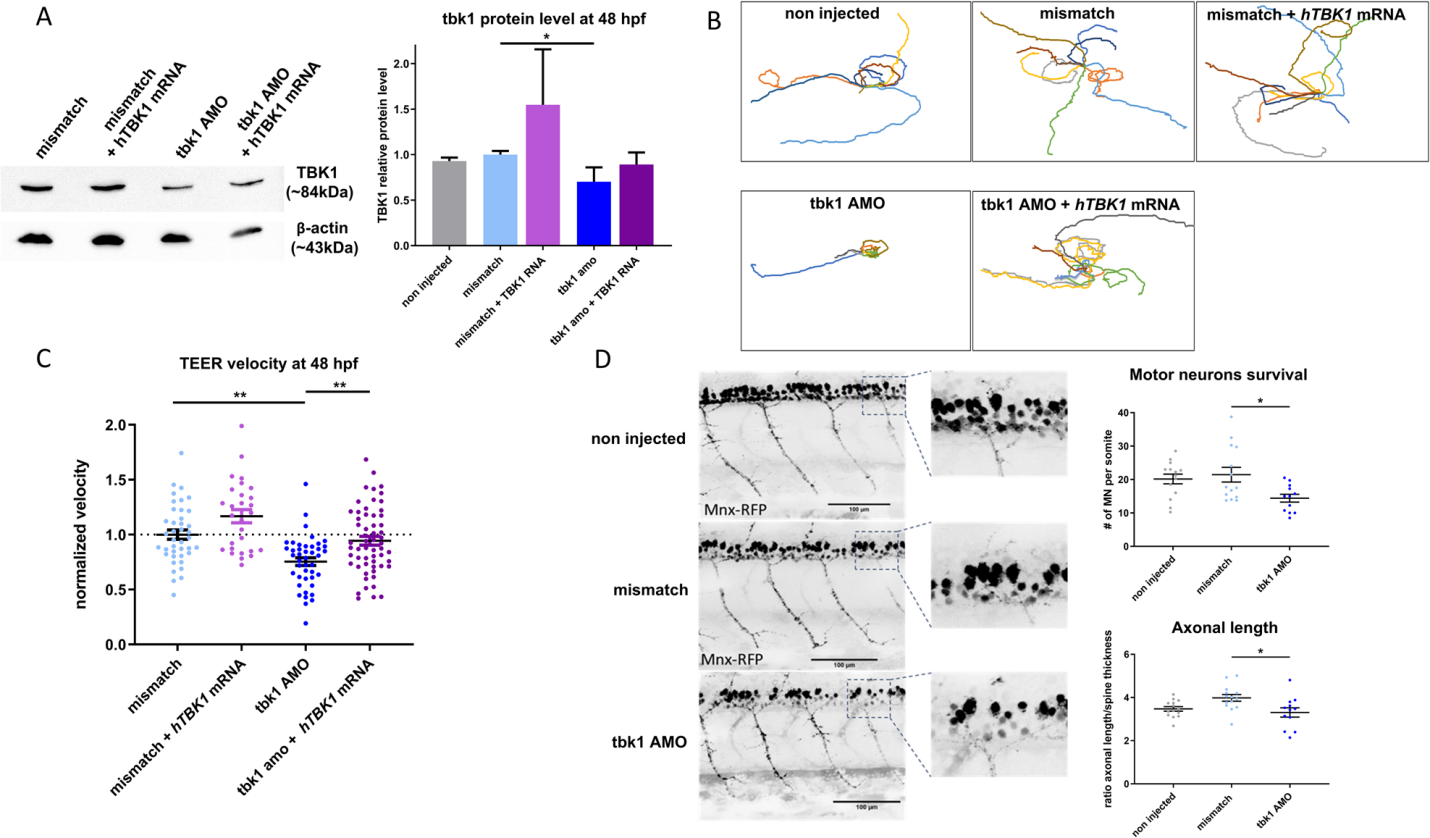
Motor behavior and motor neuron phenotype induced by tbk1 knockdown in zebrafish larvae. **A** Western blot quantification (ratio to β-actin level) of tbk1 expression after antisense morpholino oligonucleotide (AMO)- and *hTBK1* mRNA injections. **B** Traces representative of the touch-evoked escape response (TEER) in 48 hpf zebrafish injected with tbk1 or mismatch AMO and *hTBK1* mRNA injections. **C** Quantitative analysis of the TEER velocity of 48 hpf zebrafish larvae injected with tbk1 or mismatch AMO and rescued with mRNA encoding human TBK1. One-way ANOVA with Tukey’s multiple comparisons test was used. *p < 0.05, **p < 0.01). **D** In vivo quantification of spinal motor neuron axonal length and number in 48 hpf transgenic Tg(mnx1:gal4/UAS:RFP) embryos injected with tbk1 or mismatch AMO.

To investigate the effect of *tbk1* expression knockdown in zebrafish, the embryos were injected at the one-cell stage with an antisense morpholino oligonucleotide (AMO) targeting the start codon of tbk1. At 48 h postfertilization (hpf), decreased expression of tbk1 was confirmed by western blotting compared with that in mismatch control and noninjected zebrafish larvae, indicating more than 40% reduced expression and thus validating the specific knockdown of tbk1 (Fig. 1A). Co-injection with mRNA encoding the human TBK1 protein was detected by immunoblotting and resulted in partial rescue of protein level (Fig. 1A).

We analyzed the motor phenotype induced by tbk1 knockdown by recording behavior using a touch-evoked escape response (TEER) test in 48 hpf larvae which is commonly used to reflect the overall locomotor capacity of the larvae. The representative traces from TEER recordings of tbk1 AMO-injected larvae are indicative of a drastic decrease in their swimming capacity (Fig. 1B). More importantly, upon injection of the mRNA encoding human TBK1, the motor phenotype and normalized velocity of tbk1 knockdown zebrafish were rescued (Fig. 1C), confirming that the motor deficits were caused by tbk1 knockdown.

The effect of tbk1 deficiency on motor units was measured in vivo using confocal imaging of transgenic Mnx:gal4/UAS:RFP zebrafish at 48 hpf. At this stage, the total number of motor neurons was significantly decreased in the spinal cord of the tbk1-depleted larvae (Fig. 1D). Notably, axonal projections from motor neurons were equally affected under these conditions (Fig. 1D). We observed a decreased number of motor neurons as early as 32 hpf (Sup. Fig. 2). The length of muscle fibers was determined in the trunk and tail regions of tbk1 AMO-injected larvae at 3 and 5 dpf, and no significant alterations were detected (Sup. Fig. 3). Therefore, tbk1 knockdown leads to degeneration or an altered development of motor neurons without major muscle alterations.

The larvae were then incubated with the mTOR inhibitor and autophagy inducer rapamycin at a dose of 0.5 µM for 24 h. We did not observe any effect of rapamycin on the motor phenotype caused by the reduced expression of tbk1 in embryos, as measured by TEER at 48 hpf (Sup. Fig. 4).

In line with previous studies, AMO injections of zebrafish embryos led to a very mild phenotype after 5 dpf (data not shown) [23]. Thus, we further investigated the phenotypic features and pathogenic mechanisms associated with TBK1 deficiency by generating and characterizing CRISPR-Cas9-induced deletion mutants of tbk1 in zebrafish.

### *tbk1* deletion induced by CRISPR-Cas9 is responsible for motor defects and early lethality in zebrafish larvae

**Fig. 2:**
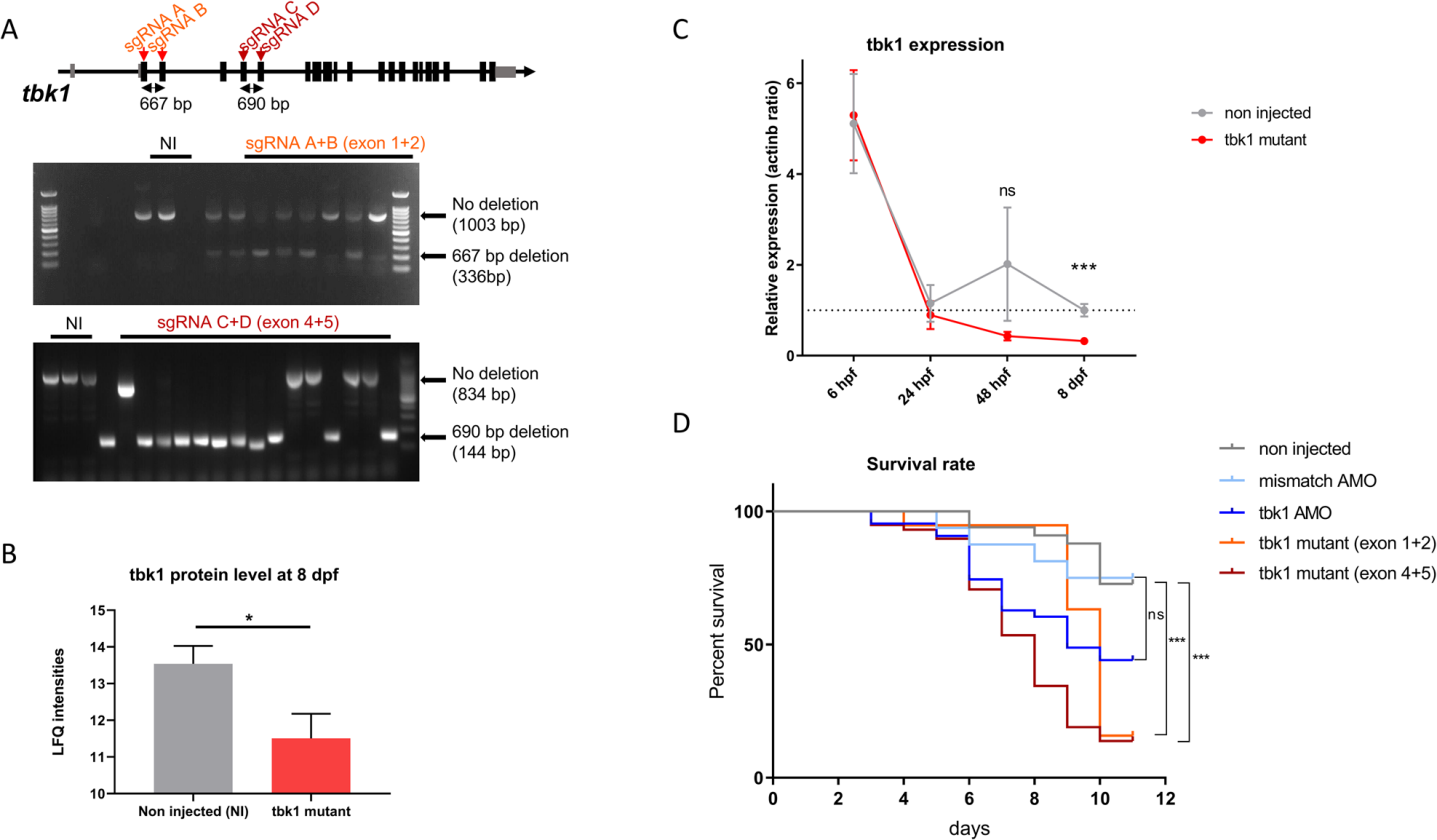
Tbk1 expression and survival in zebrafish larvae with CRISPR-induced deletion of the tbk1 gene. **A** Description of CRISPR-Cas9 tandem sgRNA targeting exons 1 and 2 or exons 4 and 5 of the tbk1 gene (top) and genotyping of injected embryos with gel electrophoresis of PCR products amplifying the deleted region. **B** tbk1 protein quantification by mass spectrometry in zebrafish larvae harboring mosaic tbk1 mutations or their noninjected controls (NIs) (unpaired t test, *p < 0.05). **C** RT‒qPCR measurement of tbk1 expression in zebrafish larvae with mosaic deletions of the tbk1 gene or NI controls between 6 hpf and 8 dpf (unpaired t test, ***p < 0.001). **D** Survival rate of zebrafish larvae injected with tbk1 or mismatch AMO (ns), CRISPR-Cas9 sgRNA targeting exons 1 and 2 or exons 4 and 5 of the tbk1 gene or NI controls (log-rank (Mantel‒Cox) test, *** *p* < 0.001).

The strategy selected to generate large deletions in the well-conserved kinase domain relies on the use of tandems of single guide RNA (sgRNA) targeting either exons 1 and 2 or exons 4 and 5 of the *tbk1* gene [24]. The deletions caused by injections of sgRNAs were validated by PCR amplification of the cDNA of zebrafish embryos. We observed approximately 70% efficacy in generating disabling large deletions, without accounting for shorter indels, in CRISPR-Cas9-injected individual embryos harboring mosaic mutations (Fig. 2A). Deletion of the tbk1 gene did not significantly affect the general larval development of zebrafish, and size measurements revealed no differences between tbk1 mutant and control larvae (Sup. Fig. 5). An internal control was established using sgRNA targeting the *tyrosinase* gene, which is essential for the early development of pigmentation in zebrafish larvae. The significant decrease in pigmentation of larval zebrafish was used as a functional validation of CRISPR-Cas9 activity in parallel (Sup. Fig. 4). The effect of CRISPR-induced deletions on the tbk1 protein level was measured by mass spectrometry at 8 dpf. Tbk1 protein levels were significantly decreased in zebrafish larvae at this stage, with up to a 60% decrease compared to the control levels (Fig. 2B). In parallel, expression was measured by RT‒qPCR at several developmental stages. A significant decrease in the mRNA level was detected at 8 dpf, with up to a 70% decrease in tbk1 expression in mutant larvae (Fig. 2C). CRISPR-Cas9 injections did not affect tbk1 expression before 48 hpf, suggesting that maternal tbk1 mRNA contributes substantially to early development (Fig. 2C). Survival to adulthood in tbk1 CRISPR-Cas9-injected fish was significantly reduced, and up to 90% mortality was observed between 9 and 13 dpf (Fig. 2D). By comparison, transient inhibition of tbk1 with AMO affected larval survival in a non-significant manner (*p* = 0.0503). This result emphasizes the key role of tbk1 in zebrafish larval development. Due to extremely high mortality at the juvenile stage we failed to establish a stable line with a characterized large deletion of the *tbk1* gene. The investigation of tbk1 function was performed using the F0-injected zebrafish model.

**Fig. 3:**
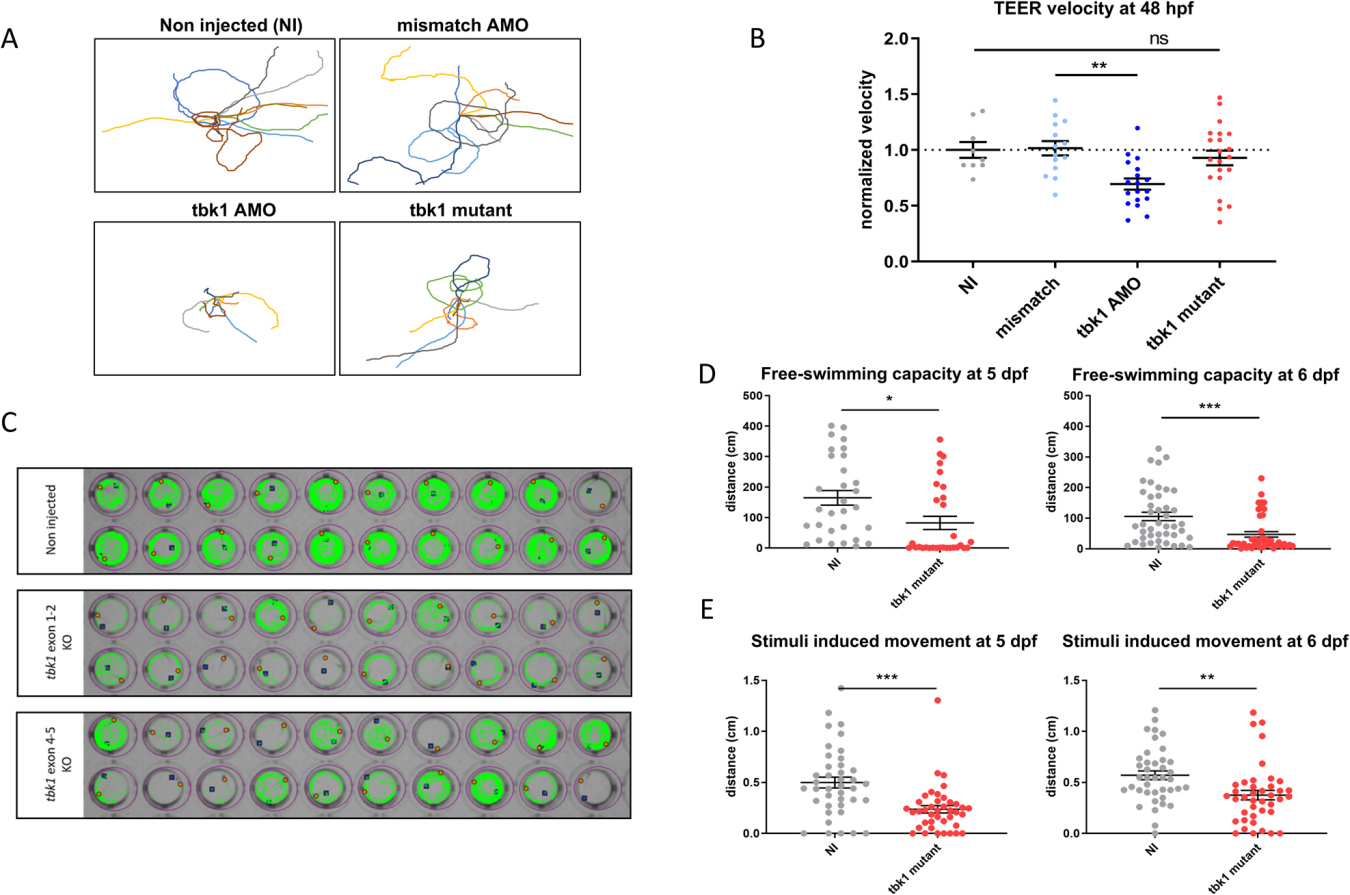
Motor behavior of tbk1 mutant zebrafish larvae. **A** Traces representative of the touch-evoked escape response (TEER) in 48 hpf tbk1 mutant, tbk1 or mismatch AMO or NI zebrafish larvae. **B** Quantitative analysis of the TEER velocity of 48 hpf tbk1 mutant, mismatch or tbk1 AMO or NI zebrafish larvae (one-way ANOVA with Tukey’s multiple comparisons test). *p < 0.05, **p < 0.01). **C** Representative traces suggestive of motor behavior and swimming trajectories of 5 dpf zebrafish larvae, recorded with a semiautomated video tracking system (Viewpoint Zebrabox). **D** Quantitative analysis of free-swimming (top panel) or stimuli-induced motor behavior (bottom) in 5 (left) or 6 dpf (right) tbk1 mutant or NI control zebrafish larvae. (Unpaired t test for comparative analysis. *p < 0.05).

Similarly, motor deficits in the tbk1 CRISPR mutant larvae were characterized using the TEER assay at 48 hpf (Fig. 3A). However, compared to zebrafish in which tbk1 was knocked down, tbk1 CRISPR mutant larvae did not show any deficits associated with motor behavior at this stage (Fig. 3B). Following the assessment of evoked swimming behavior at the early stages of development, we further analyzed spontaneous swimming behavior at 5 dpf by performing a free-swimming test of individual larvae for periods of 10 min in a 48-well plate format using the semiautomated zebrabox tracking module (Fig. 3C). Compared with that of NI controls, the total swimming distance of tbk1 mutant larvae was reduced by more than 50% at 5 and 6 dpf (Fig. 3D). A similar significant decrease in swimming capacity was observed when measuring the stimulated behavior using after an audio stimulus (Fig. 3E).

### Nicotinamide metabolism is impaired in tbk1 mutant zebrafish

**Fig. 4:**
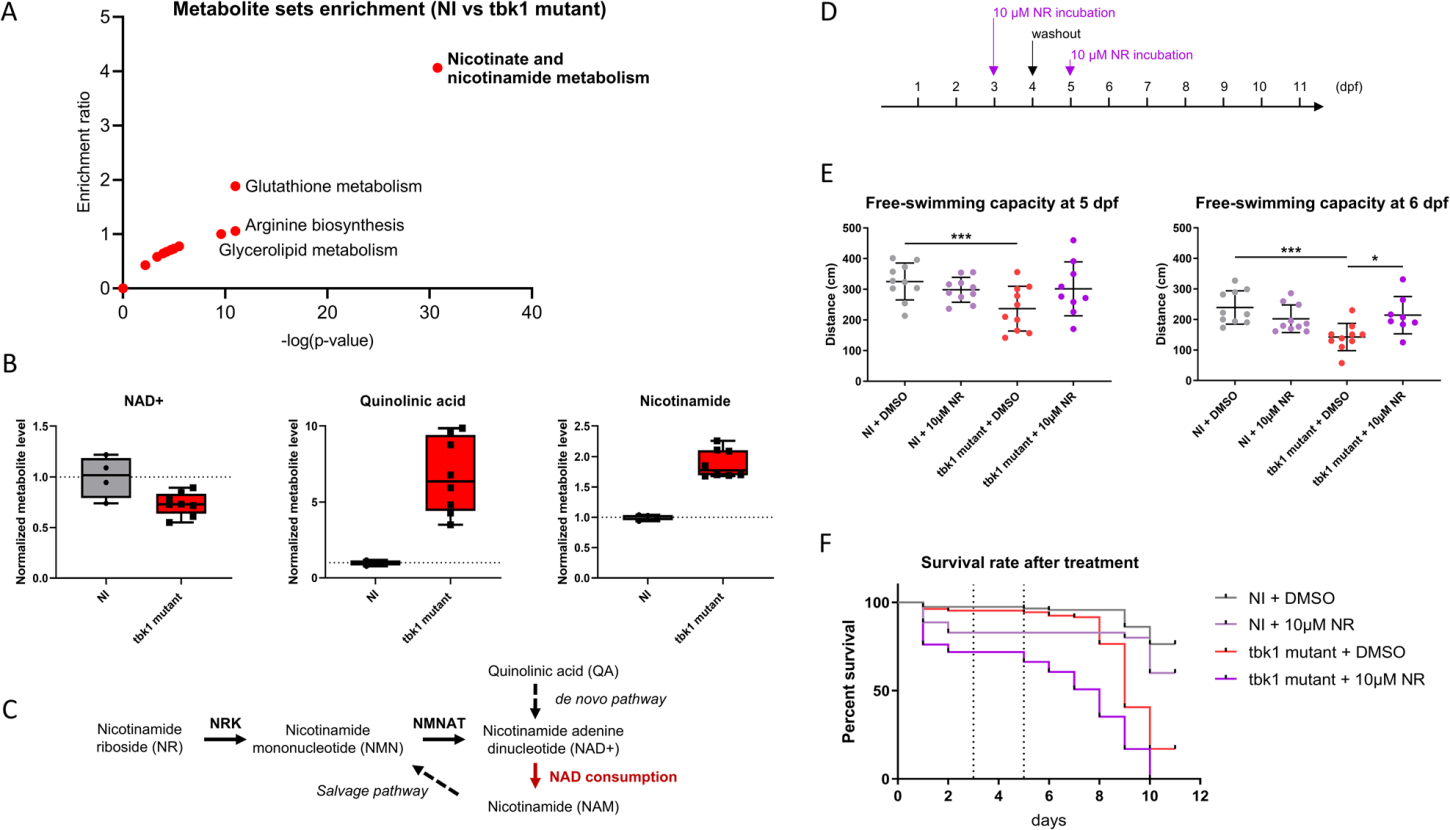
Metabolomic analysis and nicotinamide riboside treatment of tbk1 mutant zebrafish. **A** Metabolite set enrichment analysis showing the enrichment ratio and statistical significance of metabolic pathways in 6 dpf tbk1 mutant zebrafish compared to their noninjected controls (over representation analysis using Metaboanalyst 6.0 with significantly affected metabolites, *p* < 0.01). **B** Mass spectrometry quantification of NAD + , quinolinic acid and nicotinamide in tbk1 mutant or NI zebrafish (unpaired t test, *p* = 0.002, *p* < 0.001 and *p* < 0.001, respectively). **C** Schematic representation of the Nicotinamide riboside (NR) and NAD+ salvage pathway affected in tbk1 mutant zebrafish (NRK, nicotinate riboside kinase; NMNAT, nicotinamide mononucleotide adenylyltransferase). **D** Schematic timeline used for the treatment of tbk1 CRISPR-Cas9-injected or NI zebrafish with 10 µM NR or 0.1% DMSO in zebrafish embryo water. **E** Quantitative analysis of larval free-swimming motor behavior upon incubation with NR (one-way ANOVA with Tukey’s multiple comparisons test). *p < 0.05, ***p < 0.001). **F** Survival rate of tbk1 mutant or NI larvae upon incubation with NR.

Considering the pleiotropic effects associated with TBK1 deletion and the common metabolic alterations associated with motor neuron defects, we hypothesized that the levels of several metabolites would be affected in tbk1 mutant zebrafish and performed a metabolomic analysis of whole larval tissues at 6 dpf before the onset of severe mortality. Metabolic profiling by mass spectrometry allowed the identification of 131 metabolites, 30 of which were significantly different. The *p* value rankings of the differentially enriched metabolites revealed glycerol 3-phosphate (G3P), nicotinamide (1.8-fold, NAM), quinolinic acid (6.5-fold, QA), and nicotinamide adenosine dinucleotide (0.7-fold, NAD + ) as the top metabolites respectively. Pathway enrichment analysis confirmed strong dysregulation of 3 out of 5 compounds in the nicotinate and nicotinamide metabolism pathway (*p* < 0.05) (Fig. 4A), with increased levels of NAM and QA and a decreased level of NAD+ in *tbk1* mutant larvae compared to their WT siblings (Fig. 4B). The same experiment was performed on 8 dpf mutant larvae and revealed an impairment of the nicotinamide metabolism similarly (top term from enrichment analysis, data not shown), confirming the persistence of this alteration after the onset of the more severe phenotype.

To validate this finding, we investigated the potential rescue of the NAD metabolism defect by treating *tbk1* mutant larvae with the NAD precursor nicotinamide riboside (NR) (Fig. 4C). The tbk1-deficient larvae were incubated with 10 µM NR after 3 dpf with a 24-hour washout to alleviate compound toxicity, and their motor behavior was recorded at 5 and 6 dpf with the Zebrabox module (Fig. 4D). The swimming behavior measurements revealed a significant rescue of the free-swimming capacity of the mutants at 6 dpf (Fig. 4E). Notably, incubation of larvae with NR was not sufficient to rescue the impaired survival of tbk1-deficient zebrafish (Fig. 4F).

### *tbk1* deletion in zebrafish causes increased necroptosis

**Fig. 5:**
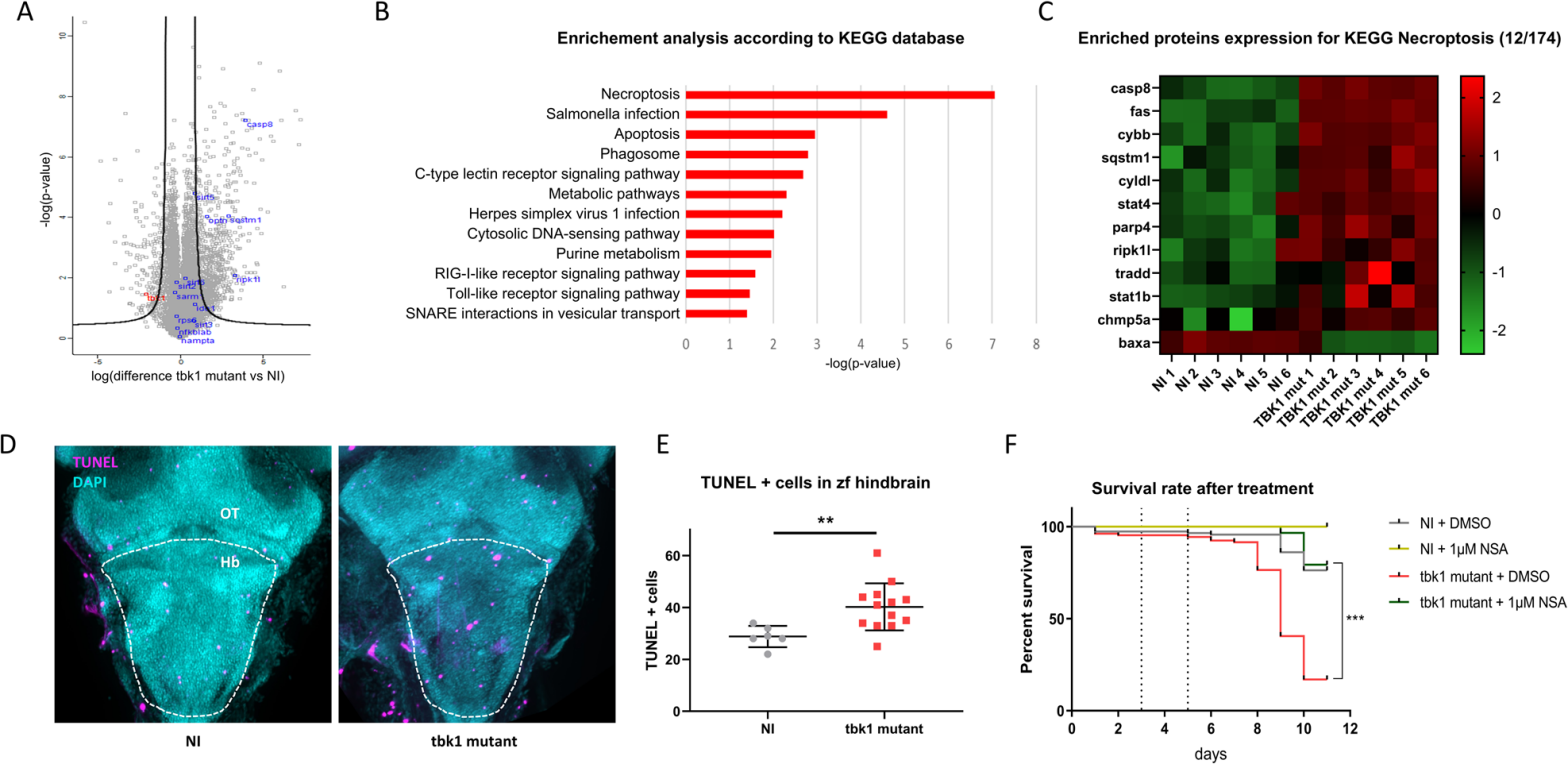
Proteomic profile and markers of programmed cell death in tbk1 mutant zebrafish. **A** Volcano plot showing protein enrichment measured by mass spectrometry in tbk1 mutant zebrafish at 8 dpf compared to their NI controls (black line = cutoff for statistical significance). **B** Pathway enrichment analysis based on KEGG 2019 for significantly enriched proteins measured in tbk1 mutant zebrafish compared to their NI controls. **C** Heatmap representation of protein levels (row z scores) for significantly enriched proteins associated with the KEGG term necroptosis (hsa04217) in tbk1 mutant and NI zebrafish larvae. **D** Terminal deoxynucleotidyl transferase dUTP nick end labeling (TUNEL) staining of cells affected by programmed cell death in the larval brain of tbk1 mutant or NI zebrafish at 6 dpf (OT, optic tectum; Hb, hindbrain; dotted line = outline of hindbrain used for quantification). **E** Quantification of TUNEL-positive cells in the hindbrain of tbk1 mutant or NI zebrafish (unpaired t test, *p* = 0.0098). **F** Survival rate of tbk1 mutant or NI larvae upon incubation with the necroptosis inhibitor necrosulfonamide (NSA) at 1 µM or 0.1% DMSO (log-rank (Mantel Cox) test). ***p < 0.001).

Proteomic analysis was performed to decipher the molecular mechanisms associated with the onset of severe behavior in 8 dpf tbk1-deficient larvae. Proteomic analysis revealed 8640 annotated proteins, 326 of which were significantly dysregulated in the tbk1 mutant group compared to the WT control group after ANOVA with FDR adjustments. Significant downregulation of tbk1 expression was confirmed, and gene set enrichment analysis was used to identify dysregulated pathways (Fig. 5A). According to the KEGG annotation database, several pathways associated with innate immunity, including Salmonella infection, the C-type lectin receptor signaling pathway, HSV1 infection, the cytosolic DNA sensing pathway, the RIG-I-like receptor signaling pathway and the Toll-like receptor signaling pathway, were significantly impaired (Fig. 5B). Most importantly, a very significant dysregulation of genes associated with apoptosis and necroptosis was observed, with upregulation of the expression of the zebrafish CASP8 and RIPK1 orthologs, suggesting a major role for necroptosis in the severe phenotype caused by tbk1 deletion (Fig. 5A–C). A detailed comparison of enriched proteins associated with necroptosis revealed an upregulation of 11 of the 12 terms differentially expressed in this pathway in tbk1 mutant zebrafish compared to NI controls (Fig. 5C). We performed TUNEL staining of whole larvae at 8 dpf and observed an increased number of TUNEL-positive cells in the hindbrains of tbk1-deficient zebrafish compared to those in the hindbrains of their noninjected WT siblings (Fig. 5D, E). This observation is in accordance with the potential key role of TBK1 in regulating the programmed cell death response in the CNS. Finally, we tested the ability of the necroptosis inhibitor necrosulfonamide (NSA) to mitigate the severe phenotype and mortality associated with tbk1 deficiency in zebrafish. We noted a promising rescue of the impaired survival in mutant larvae incubated with 1 µM NSA after 3 dpf (Fig. 5F).

### The expression of proteins associated with the ALS pathway is affected in tbk1 mutant zebrafish and iPSC-derived motor neurons from ALS patients with TBK1 mutations

**Fig. 6:**
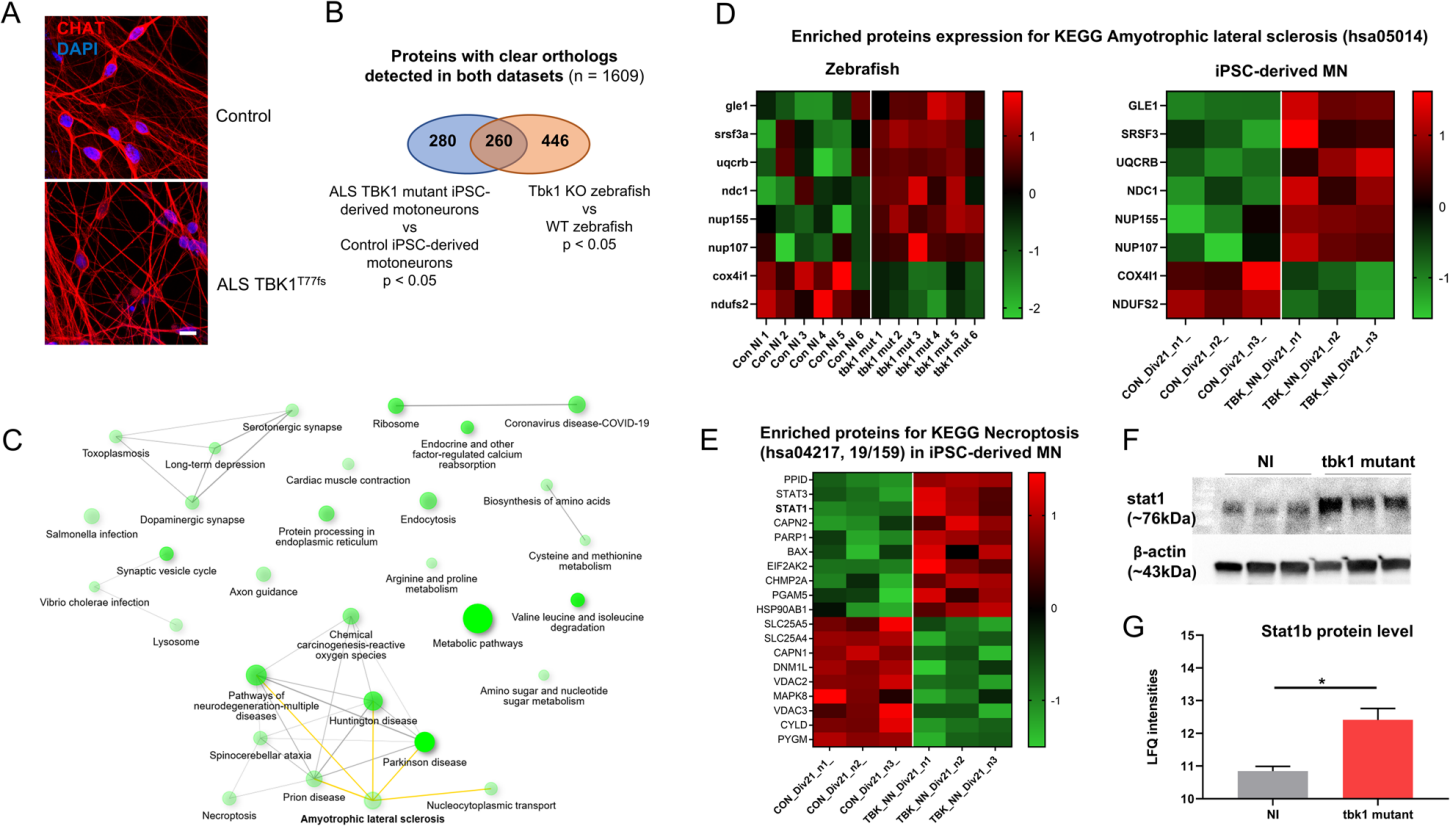
Joint proteomic analysis of tbk1 mutants zebrafish and iMNs from ALS patients with TBK1 mutations. **A** Fluorescent immunolabeling of the neuronal marker CHAT in differentiated induced pluripotent stem cell-derived motor neurons (iMNs) at day 21 from an ALS patient harboring the loss of function mutation TBK1^T77fs^ compared to control iMNs. **B** Proteomic profiles of ALS-TBK1 iMNs compared to control iMNs. The differentially expressed protein list was compared to the list of zebrafish orthologs differentially expressed in tbk1-deficient zebrafish larvae, and enrichment analysis was performed on the common list of 260 terms. **C** Network plot showing the relationship between enriched pathways from TBK1-mutant ALS models. A close interaction between the necroptosis pathway and several neurodegenerative pathways, including the amyotrophic lateral sclerosis (ALS) KEGG pathway, is observed. **D** Heatmap representation of protein levels (row z scores) for significantly enriched proteins in both datasets associated with the KEGG term ALS in tbk1 mutant vs NI zebrafish and ALS-TBK1 iMNs vs control iMNs. **E** Heatmap representation of protein levels (row z scores) for significantly enriched proteins associated with the KEGG term necroptosis in ALS-TBK1 iMNs vs control iMNs. **F** Western blotting of STAT1 expression in tbk1 mutant and NI zebrafish. **G** Mass spectrometry quantification of stat1b protein level in tbk1 mutant and NI zebrafish.

To confirm the translational potential of our findings in the tbk1-deficient zebrafish model, we investigated iPSC-derived motor neurons obtained from an ALS patient harboring a TBK1 loss of function mutation. Similarly, proteomic analysis was performed to compare TBK1 mutant motor neurons to non-ALS control motor neurons, after 21 days of differentiation, which express the differentiated MN marker choline acetyltransferase (Fig. 6A). We compared the list of proteins with clearly identified orthologs in zebrafish from both the human motor neuron and zebrafish comparisons and identified 260 commonly dysregulated proteins (Fig. 6B). Pathway enrichment analysis revealed alterations in pathways associated with several neurodegenerative processes, including the KEGG ALS pathway (hsa05014, Fig. 6C). Among the terms associated with this pathway, GLE1, NDC1, NUP107, NUP155 and SRSF3, which target nuclear-cytosolic transport, were found to be upregulated in both cellular and animal models of ALS (Fig. 6D). An enrichment for the KEGG pathway Nucleocytoplasmic transport was identified (Fig. 6C) and supports a possible link between this pathway and the phenotype associated with TBK1 deficiency.

In line with previous findings, a significant enrichment of proteins associated with necroptosis was observed in the common list, and a similar dysregulation of 19 terms (including STAT1) was observed in the TBK1 mutant iMN (Fig. 6E). Notably, proximity between the dysregulated necroptosis and neurodegenerative pathways was suggested, as indicated by links between pathways (representing at least 20% of proteins shared between terms) (Fig. 6C). Finally, western blot quantification of the STAT1 orthologs in zebrafish shows a non-significant increase of the protein level (Fig. 6F), in line with the increased protein abundance measured by mass spectrometry in tbk1 mutants compared to their WT relatives (Fig. 6G).

## Discussion

This study highlights the requirement of tbk1 for the maintenance of motor neuron function. We showed that the downregulation of tbk1 expression using the AMO affects the viability of motor neurons and that the motor response is altered early on. Using CRISPR-Cas9, we demonstrated that deletion of the tbk1 gene leads to a severe phenotype and reduced survival in larval zebrafish. Through unbiased approaches, this study identified nicotinamide metabolism and necroptosis pathways as critical, and previously unexplored, aspects of the pathology associated with tbk1 deficiency in zebrafish. Finally, proteomic analysis of TBK1 iPSC-derived motor neurons from ALS patients validated the relevance of these findings and the translational potential of such models for future studies and drug screening.

To our knowledge, this represents the first study in which the AMO and CRISPR-Cas9 strategies were used and compared for the characterization of TBK1-ALS models. Interestingly, we observed striking differences between the phenotypes resulting from AMO knockdown in tbk1 zebrafish larvae and those resulting from CRISPR-Cas9-induced *tbk1* deletion. The mRNA levels measured in fertilized embryos suggest a strong influence of maternal mRNA on the phenotype early during development, which could explain the absence of a phenotype in CRISPR mutants before 4 dpf.

We were able to demonstrate that tbk1 display a key role in development, as shown by the high mortality of tbk1 KO zebrafish larvae, which further corroborates previous results showing that homozygous Tbk1 inactivation causes embryonic death in mice. This study in mice delineates the strong relevance of necroptosis in the priming of a developmental defect causing embryonic death. More recently, in mice harboring the p.E696K TBK1 mutation, with a selective loss of OPTN activity, it was found that autophagolysosomal dysfunction but not necroptosis was the major trigger for neurodegeneration [25]. Moreover, discrepancies have been reported regarding the importance of necroptosis in ALS pathology and no clear conclusions can be drawn [26]. In the SOD1(G93A) transgenic mouse model of ALS, RIPK1 activity was central in axonal degeneration while others found no benefit in its blocking [27–29]. Here, we show that the decrease in TBK1 activity leads to necroptosis and the initiation of a strong motor phenotype during larval development. This finding emphasizes the promising therapeutic potential of drugs targeting the necroptosis pathway. Notably, small molecule inhibitors of RIPK1 activity are currently being investigated in clinical trials to treat ALS and other neurodegenerative disorders, and promising results have been reported [30].

As mentioned previously, TBK1 deficiency has been largely examined in different contexts, including models of ALS. In this regard, autophagy defects appear to be a central consequence of downregulated TBK1 activity, as in ALS patient iPSC-derived motor neurons, although efforts to modulate autophagy have not yielded satisfying results in this context [12]. Here, we reported the absence of an effect of the mTOR inhibitor rapamycin on the motor behavior of tbk1 AMO-knockdown zebrafish larvae. We observed increased levels of autophagy cargos and receptors, including the p62 ortholog (sqstm1) and optn, suggesting a modest dysregulation of the autophagic response (enrichment for KEGG term Phagosome; adj. p val. = 0.07697) when comparing the proteomes of the WT and tbk1 mutant larvae. Although motor neuron degeneration occurs upon tbk1 knockdown, we were unable to determine the effect of tbk1 inactivation on the formation of protein aggregates in motor neurons in this model.

Finally, NAD+ depletion and alterations in nicotinamide metabolism have been observed in major neurodegenerative disorders including ALS [31]. Notably, in our zebrafish model, we observed an increased level of the neurotoxic intermediate quinolinic acid, which has been shown to accumulate and play a role in ALS pathogenesis [32]. NAD+ precursors such as NR have been shown to protect motor neurons and elicit beneficial effects in several models of ALS in vitro and in vivo, as well as in models of Wallerian degeneration or other neurodegenerative diseases [33]. The motor behavior rescue observed in our zebrafish model reinforces the potential of such metabolic intervention and establishes a link between impaired TBK1 activity and the depletion of NAD + . Although we do not provide a mechanistic explanation for this link, the modulation of NAD+ levels by TBK1 has previously been demonstrated through the activity of cGAMP and the kinase ATM [34]. A recent study revealed that impairment of mitochondrial microdomains promoted proteostatic stress in a TBK1-inactivated model of ALS [35]. One could hypothesize that such alterations could be linked to the metabolic stress observed in our model.

We recognize that the use of larval zebrafish as a model in this study is a major limitation, mitigating the clinical relevance of our findings. Nonetheless, the idea of a presymptomatic phase and initial motor neuron hit prior to clinical onset is commonly disregarded in animal models, and zebrafish models have proven to be valuable in this regard. Furthermore, we analyzed the proteome of patient-derived cells and used a comparative approach to validate the translational potential of this model. By specifically comparing the protein content of whole larval tissues and cellular extracts from motor neurons, we were able to identify clear ALS-specific mechanisms common to both zebrafish and human TBK1 mutant models. Why the observed molecular signature is associated with enriched markers of the nucleocytoplasmic export machinery remains to be clearly established.

Based on the proteomic dataset comparison, the enrichment of the STAT1 ortholog stat1b was measured in tbk1 mutant zebrafish, and similarly, STAT1 was found to be enriched in TBK1 mutant motor neurons. STAT1 is a key transcription factor involved in the cellular response to IFN which is well conserved between teleosts and mammals [36]. STAT1 activation upon lipopolysaccharide stimulation was reported to be strongly inhibited in TBK1-deficient macrophages [37]. Furthermore, C9ORF72 deficiency was shown to cause enhanced inflammation through hyperactivation of STAT1 and STING, with increased protein levels [38]. Interestingly, STAT1 signaling has been shown to reduce axonal degeneration by activating the NAD+ metabolic pathway [39]. Therefore, the observed upregulation of STAT1 could indicate an inadequate response to immune stimulation and considering its role in the regulation of cell death through NAD+ metabolic signaling, this signaling pathway could represent an attractive therapeutic target.

Taken together, these results indicate a substantial contribution of tbk1 activity to the normal development of motor neurons. Nicotinamide metabolism and necroptosis were identified as critical elements of tbk1 deficiency, thus providing new insights into the molecular and metabolic changes that could precipitate the death of motor neurons. Last, these findings pave the way for further studies focusing on the innate immune response in ALS, and the models described here should prove to be efficient tools for future drug screening.

## Materials and methods

### Ethics approval and consent to participate

Adult and larval AB wild-type zebrafish (*Danio rerio*) were maintained at the Institut Imagine, Paris, zebrafish facility, in compliance with the French Institutional Animal Care (CEEA-LR-13007) and bred according to the National and European Guidelines for Animal Welfare. All procedures were approved by the Institutional Ethics Committees at the Research Centers of Imagine. All procedures with human material were in accordance with the ethical committee of the Ulm University (Nr.0148/2009 or 265/12) and in compliance with the guidelines of the Federal Government of Germany. The use of human material was approved by the Declaration of Helsinki concerning Ethical Principles for Medical Research Involving Human Subjects.

### Experimental model maintenance and handling

The embryos were raised at 28 °C and kept in egg water (0.06 g/L aquarium salt (Instant Ocean, Blacksburg, VA) in Milli-Q water +0.01 mg/L methylene blue) until 5 days post fertilization. At 5 days postfertilization, the embryos were transferred to water. Egg clutches were collected from a single cross and injected at the one-cell stage. In this study, both male and female fish were used. The following transgenic lines were used for imagery: Tg(*Mnx:Gal4/UAS:RFP)*.

### Morpholino and sgRNA synthesis and injection

Microinjections of antisense morpholino oligonucleotide (AMO) were performed on wild-type and *Mnx:RFP* transgenic AB fishes at the one-cell stage using a PV 820 Pneumatic PicoPump injector (World Precision Instruments, USA) and heat-pulled borosilicate glass needles. *tbk1* morpholino (synthesized by Gene Tools, Philomath, USA) targeting the initial ATG codon of the *tbk1* gene (AMO-tbk1: 3’-ggccgtactctgcatgatgactgta -5’) was injected at the one-cell stage to achieve transient knockdown of *tbk1*. A mismatch control morpholino bearing a nucleotide sequence (mismatch 5’-cctcttacctcagttacaatttata-3’) that does not bind any zebrafish mRNA was used to exclude any injection-related phenotype. Microinjections were performed at 0.45 mM for both the *tbk1*-targeting morpholino and the mismatch control.

The target nucleotide sequences for guide RNA design were selected using CHOPCHOP software and a scoring method [40], and the sequences were obtained via direct synthesis of RNA oligonucleotides (Alt-R® CRISPR-Cas9 crRNA, IDT, USA). The sequences are listed below:

sgRNA *tbk1* exon 1: GCCACAGCUAACGUGUACCG

sgRNA *tbk1* exon 2: CGUCAAGCUGUUCGCCGUCG

sgRNA *tbk1* exon 4: AUGAAUCACCUGCGCGAGUA

sgRNA *tbk1* exon 5: CUGCCGUUCAGACCCUUCGA

sgRNA *tyrosinase* exon 1_1: CAGUAUCCUCACUCAGGAGU

sgRNA *tyrosinase* exon 1_2: UCCAGCUGUCCACUACCGAG

Guide RNAs were combined with a Cas9 recruitment sequence (Alt-R® CRISPR-Cas9 tracrRNA, 1072532, IDT, USA) by mixing 2 μL of each type of nucleotide at 50 μM and incubating for 5 min at 95 °C. To prevent Cas9 precipitation, 0.4 μL of HEPES buffer was added (200 mM HEPES-NaOH pH 7.5, 1.5 M KCl). To induce larger deletions in the *tbk1* gene, paired guide RNAs were used. The injection solution was prepared by mixing 1 μL of each guide solution with 2 μL of the Cas9 enzyme (Alt-R® S.p. Cas9 Nuclease V3, 1081058, IDT, USA) to obtain a final volume of 4 μL. Microinjections were performed on wild-type or Tg(*Mnx:Gal4/UAS:RFP)* AB fishes at the one-cell stage using a PV 820 Pneumatic PicoPump injector (Wold Precision Instruments, USA) and heat-pulled borosilicate glass needles.

### PCR amplification

To assess the correct deletion induced in the *tbk1* gene, PCR amplification of the target region was performed. For each injection set, DNA extraction from 20 larvae was performed through incubation in 40 μL of extraction buffer (25 mM NaOH, 0.2 mM EDTA) for 1 h at 95 °C, followed by the addition of 40 μL of Tris-HCl (40 mM, pH 5.5). The solution was stored at 4 °C until further manipulation. PCR amplification was performed using the following tbk1 primers (Eurofins, USA):

*tbk1* exon 1–2 deletion: F 5’-TCTGAAGCTCATGCCTGTGTG-3’;

R 3’-CCTGAGAAGAGCTGCTGGATTA-5’

*tbk1* exon 4–5 deletion: F 5’-ACACATCACATTCACTCAAAACTAA-3’;

R 3’-GCTCGTCATGCTAACTCCACT-5’

For each sample, 2 μL of DNA was mixed with 10 μL of Hot Start Taq 2X Master Mix (NEB, M0496), 0.8 μL of each primer and 6.4 μL of sterile water. The PCR amplification program was optimized for 30 cycles at an annealing temperature of 60 °C. A 1% migration gel was prepared using agarose (A9539, Sigma Aldrich) in 1X TAE (A4686, PanReac AppliChem).

### Locomotion analysis of zebrafish embryos

Locomotor activity was assessed at 48 h postfertilization through a touch-evoked escape response test. Larvae were placed at the center of a 15 cm Petri dish, and their tails were lightly touched with a tip. This stimulus provoked an escape response that was recorded using a grasshopper digital camera (Point Grey) at a speed of 30 frames/second. Larvae were scored for distance swam, travel time and velocity using the Fiji plugin manual tracking.

At later stages (>3 dpf), locomotor activity was assessed using a spontaneous locomotion test. Vitality, capacity to swim and normal development of larvae was controlled under magnifying lens in a petri dish before they were accustomed and maintained in a 96-well plate, and their total locomotor activity was recorded using a Zebrabox (Viewpoint Life Sciences, Lyon, France). The protocol used was composed of 2 distinct periods: 1) 40 min of free swimming without any stimulus (dark environment) and 2) 2 stimuli separated by a 10-minutes period to avoid any habituation effect. Each stimulus consisted of a 1 second sound (450 Hz, 80 dB) and exposure to bright light. Total swim activity was analyzed using ZebraLab V3 software © (Viewpoint Life Sciences, Lyon, France). The escape response induced by the stimuli was quantified by measuring the distance swam 5 seconds after stimulation.

### Whole-mount zebrafish larvae imaging and muscle-axonal measurements

At 50 hpf, Mnx-RFP larvae were anesthetized with tricaine and fixed in 4% FA for 30 min at 4 °C and agitated. Larvae were then washed 3 times in 1X PBS at 4 °C. The larvae were mounted by removing the cephalic part in a 70% glycerol (G9012, Sigma‒Aldrich) solution. Images were taken with an apotome (Apotome.2, Zeiss) at 20x magnification and a laser of 561 nm (HXP 120 V, OSRAM) for spinal integrity investigation or bright light for muscle fiber measurements.

### TUNEL staining

Zebrafish larvae aged 8 dpf were deeply anesthetized with tricaine, fixed in 4% FA for 30 min at 4 °C, and agitated. Larvae were then washed in PBS and dehydrated in methanol using increasing concentrations of 25%, 50%, and 75% in PBS. Larvae were stored in 100% methanol at −20 °C for at least 1 h before rehydration with decreasing methanol concentrations and washing in PBS. Proteinase K digestion was performed for 90 min at 37 °C. Larvae were postfixed in 4% FA for 15 min and washed again in PBS. Apoptotic cells were stained using the Click-iT Plus TUNEL Assay, and larvae were first incubated with terminal deoxynucleotidyl transferase before retraining and incubation with the Click-iT™ Plus TUNEL reaction cocktail. DNA counterstaining was performed using DAPI. Whole larvae were mounted in glycerol for imaging using a spinning disk system (Zeiss, Germany). Image analysis and cell counting were performed using ImageJ software.

### Immunoblotting

Thirty micrograms of protein from zebrafish lysates (an equal amount of protein in each lane) was separated by sodium dodecyl sulphate polyacrylamide gel electrophoresis. The samples were denatured at 98 °C for 7 min. The separated proteins were transferred to nitrocellulose membranes (0.45 μm, Life Sciences) and probed with the following primary antibody: NAK1/TBK1 antibody (ab109735, Abcam), STAT1 (D1K9Y) antibody (#14994 Cell signaling) and α-tubulin antibody (T5168, Sigma‒Aldrich) was used as a loading control. The blots were incubated with the corresponding fluorescent secondary antibody, and the signal was detected using the ODYSSEY® CLx. The intensity of the bands in each of the lanes from the Western blots was measured by ImageJ.

### Drug incubation and survival measurement

Groups of 12-10 larvae were kept in 12-well multidisks in 3.5 mL of either egg water (*0.06* *g/L aquarium salt (Instant Ocean, Blacksburg, VA) in Milli-Q water* + *0.01* *mg/L methylene blue*) at 28.5 °C from 24 hpf to 5 dpf or water after 5 dpf. Larvae were fed daily with paramecia, and the media was changed every two days. When needed, media were supplemented with 10 μM caffeic acid (C0625, Sigma‒Aldrich) or nicotinamide riboside (SMB00907, Sigma‒Aldrich), and dimethyl sulfoxide was used as a negative control (D2438, Sigma‒Aldrich). The number of dead larvae was scored each day for 12 days.

### MNs differentiation from human iPSCs

Human iPSC lines from a 41-years old male ALS patient carrying the *TBK1* c.358+2 T > C loss of function mutation (p.T77Wfs∗4) and a 64-years old male control characterized elsewhere [12] were used for motor neuron differentiation. MNs were differentiated from hiPSCs using the protocol published by Shimojo and collaborators with minor modifications, as described elsewhere [12, 41]. In brief, the following cells were detached and cultivated in suspension in ultralow attachment T75 flasks (Corning, 3815) for 3 days for the formation of EBs in hESC media: DMEM-F12 (Gibco, 31331-028), 20% KnockOutTM (Gibco, 10828028) for serum replacement, 1% NEAA, 1% β-mercaptoethanol, 1% antibiotic-antimycotic, 10 µM SB-431542 (Stemcell Technologies, 72232), 1 µM dorsomorphin (Tocris, 3093), 3 µM CHIR 99021 (Stemcell Technologies, 72054), 1 µM pumorphamine (Miltenyi Biotec, 130-104-465), 150 µM vitamin C, 500 µM cAMP (Sigma‒Aldrich, D0260), 1% Neurocult supplement (Stemcell Technologies, 05731), and 0.5% N2 supplement (Gibco, 17502-284). On the fourth day, the medium was switched to MN medium supplemented with DMEM/F12 (Gibco, 31331-028), 24 nM sodium selenite (Sigma‒Aldrich, S5261), 16 nM progesterone (Sigma‒Aldrich, P8783), 0.08 mg/mL apotransferrin (Sigma‒Aldrich, T2036), 0.02 mg/mL insulin (Sigma‒Aldrich, 91077 C), 7.72 μg/mL putrescine (Sigma‒Aldrich, P7505), 1% NEAA, 1% antibiotic-antimycotic, 50 mg/mL heparin (Sigma‒Aldrich, H4783), 10 μg/mL of the neurotrophic factors BDNF (Peprotech, 450-02), GDNF (Peprotech, 450-10), and IGF1 (Peprotech, 100-11), 10 µM SB-431542, 1 µM dorsomorphin, 3 µM CHIR 99021, 1 µM pumorphamine, 150 µM vitamin C, 1 µM retinoic acid, 500 µM cAMP, 1% Neurocult supplement, and 0.5% N2 supplement. After 5 d of cultivation, EBs were dissociated into single cells with Accutase (Sigma‒Aldrich, A6964) and plated onto μDishes (Ibidi, 81156), μPlates (Ibidi, 89626) or 6-well plates (Corning, 353046) previously coated with Growth Factor Reduced Matrigel (Corning, 356231). MNs were further cultivated for up to day 21 before immunohistochemical and proteomic analysis.

### Immunohistochemistry

Immunochemical analysis was performed as previously described [12], MNs were fixed by using 4% paraformaldehyde (Sigma-Aldrich, P6148) and 10% sucrose (Carl Roth, 4621.1) in PBS (Gibco, 14190-094). After fixation, cells were incubated for 2 h with blocking-permeabilization solution (PBS + 10% goat serum [Millipore, S26-100ML] + 0.1% Triton X-100 [Roche, 10789704001]), and the same solution was used for incubation with Anti-CHAT, 1:500 (Abcam, ab181023) (48 h at 4 °C). Afterwards cells were washed with PBS, incubated with the Alexa Fluor ^TM^ 568 goat anti-rabbit IgG (Invitrogen, A32723) secondary antibody (diluted 1:1000 in PBS), washed again and mounted with ProLong Gold Antifade reagent with DAPI (Invitrogen, P36935).

### Metabolomics analysis

Zebrafish larvae were collected at 6 and 8 dpf and stored at –80 °C until analysis. After the addition of an extraction solution composed of 50% methanol, 30% acetonitrile, and 20% water (G.M. Mackay et al., 2015) at a ratio of 1 ml per 30 mg of larvae, the samples were vortexed for 5 min at 4 °C and then centrifuged at 16,000 × *g* for 15 min at 4 °C. The supernatants were collected and separated by liquid chromatography-mass spectrometry (LC‒MS) using a SeQuant ZIC-pHilic column (Millipore Sigma). The aqueous mobile-phase solvent was 20 mM ammonium carbonate plus 0.1% ammonium hydroxide solution, and the organic mobile phase was acetonitrile. The metabolites were separated over a linear gradient from 80% organic matter to 80% aqueous matter for 15 min. The column temperature was 50 °C, and the flow rate was 200 μL/min. The metabolites were detected across a mass range of 75 to 1000 m/z using a Q Exactive Plus mass spectrometer (Thermo Fisher Scientific) at a resolution of 35,000 (at 200 m/z) in electrospray ionization and polarity switching mode. Lock masses were used to ensure mass accuracy below 5 ppm. The peak areas of different metabolites were determined using Thermo Fisher Scientific TraceFinder software using the exact mass of the singly charged ion and known retention time on the HPLC column.

As a part of the routine analytical pipeline, the recommendations of the metabolomics Quality Assurance & Quality Control Consortium were applied. The routine quality controls included regular equipment maintenance (Thermo Fisher Scientific) and the use of standard operating procedures for sample extraction, storage, and analyses. General practices also included weekly test runs to ensure system stability and quality. Regarding the quality controls (QCs) in relation to this study, we used a) pooled interstudy QC, b) process and extraction blanks, c) system stability blanks, d) solvent blanks, e) long-term reference standard interlaboratory QC mix to ensure system stability, and f) samples that were blinded and loaded in randomized order. The QC analyses of the pooled samples revealed no significant differences in metabolite levels between the QCs.

Data analysis was performed with MetaboAnalyst 5.0 software (Z. Pang et al., 2021). Samples were normalized by sum, and each metabolite level was adjusted by autoscaling. PCA was performed for all the metabolites identified. Hierarchical clustering was performed using the Ward method and included all the metabolites identified.

### Proteomic analysis

#### Sample preparation

S-TrapTM micro spin column (Protifi, Huntington, USA) digestion was performed on 20 μg of whole zebrafish larval lysate according to the manufacturer’s instructions. Briefly, the samples were reduced with 20 mM TCEP and alkylated with 50 mM chloracetamide for 15 min at room temperature. Aqueous phosphoric acid was then added to a final concentration of 1.2%, followed by the addition of S-Trap binding buffer (90% aqueous methanol, 100 mM TEAB, pH 7.1). The mixtures were then loaded on S-Trap columns. Five additional washing steps were performed for thorough SDS elimination. The samples were digested with 1.5 μg of trypsin (Promega) at 47 °C for 90 min. After elution, the peptides were vacuum-dried.

#### LC MS/MS

The tryptic peptides were resuspended in 20 μL of 2% ACN and 0.1% formic acid in HPLC-grade water, and 400 ng was injected into a nanoElute (Bruker Daltonics, Germany) HPLC (high-performance liquid chromatography) system coupled to a timsTOF Pro (Bruker Daltonics, Germany) mass spectrometer. HPLC separations (Solvent A: 0.1% formic acid in water; Solvent B: 0.1% formic acid in acetonitrile) were carried out at 250 nL/min using a packed emitter column (C18, 25 cm × 75 μm, 1.6 μm) (Ion Optics, Australia) via gradient elution (2% solvent B for 1 min; 2 to 13% for 41 min; 13% to 20% for 23 min; 20% to 30% for 5 min; 30% to 85% for 5 min; and finally 85% for 5 min to wash the column). Mass spectrometric data were acquired using the parallel accumulation serial fragmentation (PASEF) acquisition method. The measurements were carried out over the m/z range from 100 to 1700 Th. The range of ion mobilities was from 0.75 to 1.25 V.s/cm2 (1/k0). The total cycle time was set to 1.16 s, and the number of PASEF MS/MS scans was set to 10.

#### MS Data Processing and Bioinformatics Analysis

Data analysis was performed using MaxQuant (version 2.0.1.0), and data were searched with the Andromeda search engine against the UniProtKB/Swiss-Prot*Danio rerio* database, which also contains the Trembl entries (release 01–2022, 46841 entries). To search for parent mass and fragment ions, we used a mass deviation of 10 ppm for the main search and 40 ppm for the main search. The minimum peptide length was set to 7 amino acids, and strict specificity for trypsin cleavage was needed, allowing up to 2 missed cleavage sites. Carbamidomethylation (Cys) was set as a fixed modification, whereas oxidation (Met) and N-term acetylation (Prot N-term) were set as variable modifications. The false discovery rates (FDRs) at the peptide and protein levels were set to 1%. Scores were calculated in MaxQuant as described previously [42]. Proteins were quantified according to the MaxQuant label-free algorithm using LFQ intensities, and protein quantification was performed using at least 1 peptide per protein. Finally, matches between runs were allowed during the analysis.

The protein group outputs from MaxQuant were then processed in Perseus (v1.6.15.0)(45). LFQ intensity values were log2 transformed, and the reverse and common contaminant hits were removed, as well as the proteins identified only by site. Next, the data were imputed to fill missing data points by creating a Gaussian distribution of random numbers with a standard deviation of 33% relative to the standard deviation of the measured values and a 2.5 standard deviation downshift of the mean to simulate the distribution of low signal values. Finally, the remaining 8484 proteins were subjected to 2-group (NI vs TBK1) Student’s t tests (volcano plot, FDR = 0.05 and S0 = 1). From this comparison, 904 proteins were found to be significantly differentially expressed.

### Statistical analysis

All data for the zebrafish experiments are represented as the average standard error of the mean (SEM), and significance was determined using the corresponding statistical test with post hoc comparisons, as specified in the legend of each figure. All analyses were performed using Prism 8.0 (Graph Pad, CA). The significance level was set at *p* < 0.05.

## Supplementary information

Original data

Supplementary Figures 1 to 5

Metabolomic analysis - Tbk1 mut vs NI

Joint proteomics TBK1 MN and tbk1 mutant zf

Proteomic analysis tbk1 mutant zf

## Data Availability

Proteomics and metabolomics data are available as electronic supplementary material.
