# Supplementary figures and images for: TBK1 is involved in programmed cell death and ALS-related pathways in novel zebrafish models
Source: Cell Death Discov. 2025 Mar 12;11:98. doi: 10.1038/s41420-025-02374-3 (PMC11903655; doi:10.1038/s41420-025-02374-3)

A

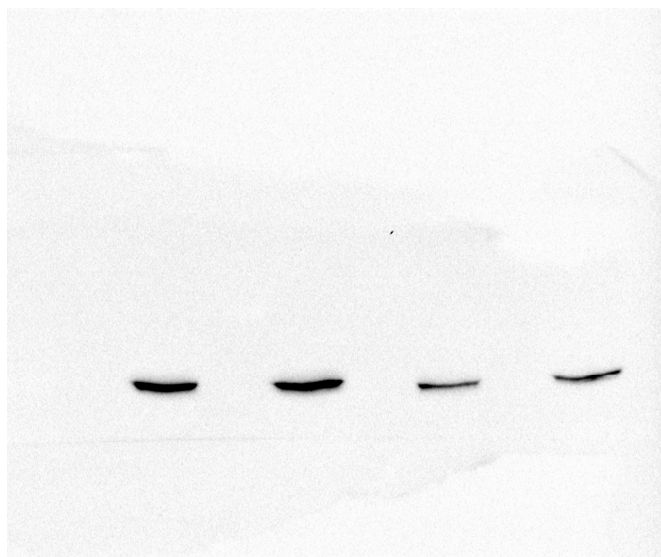

B

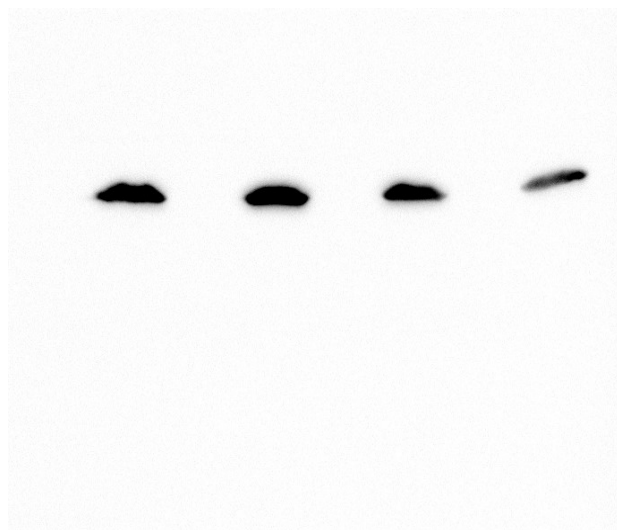

Label\_C1\_Internal

A

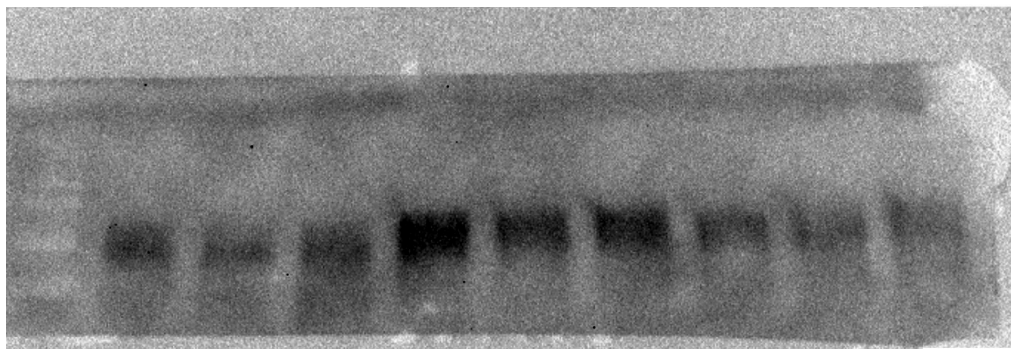

B

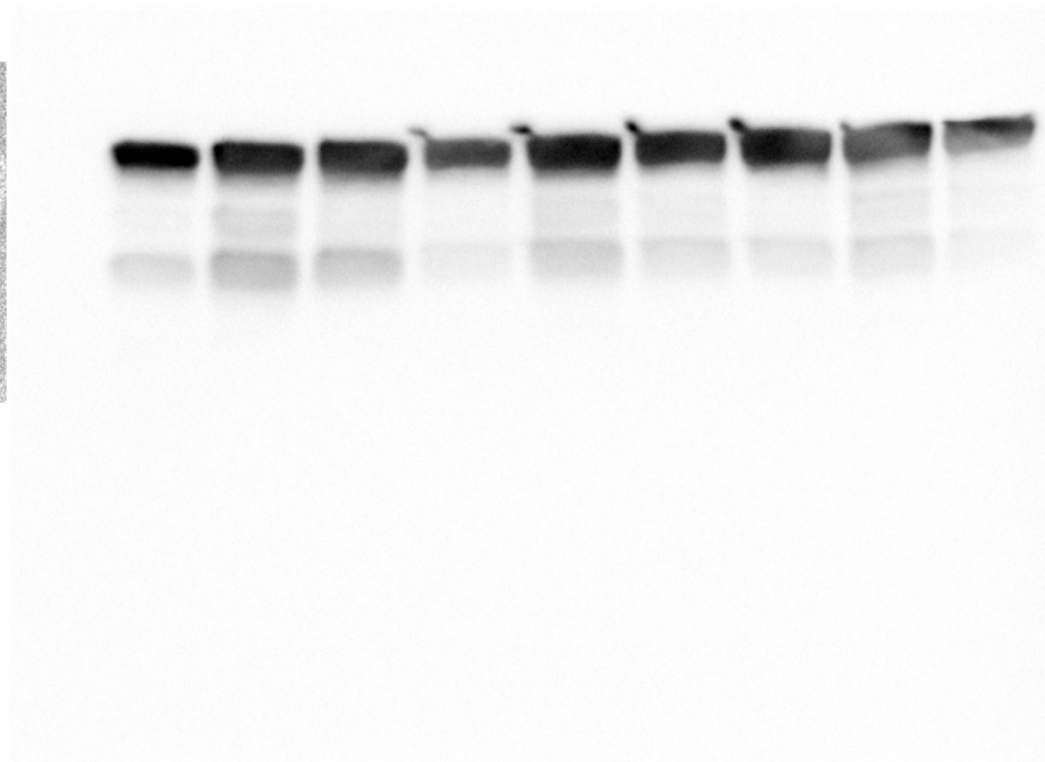

Label\_C1\_Internal

Supplement: Supplementary file 1 — Original data [file 41420_2025_2374_MOESM1_ESM.pdf]
