# Supplementary material for: TBK1 is involved in programmed cell death and ALS-related pathways in novel zebrafish models
Source: Cell Death Discov. 2025 Mar 12;11:98. doi: 10.1038/s41420-025-02374-3 (PMC11903655; doi:10.1038/s41420-025-02374-3)
Supplement: Supplementary file 2 — Supplementary Figures 1 to 5 [file 41420_2025_2374_MOESM2_ESM.pdf]

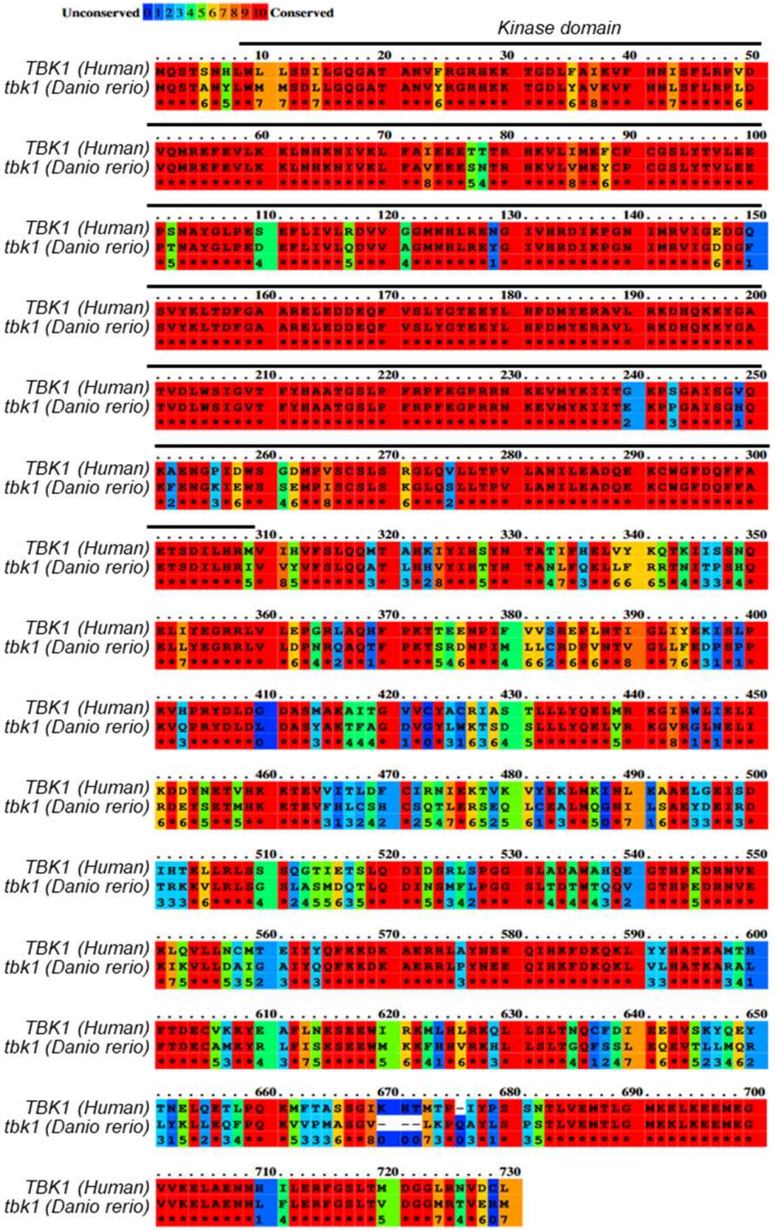
Unconserved
Conserved
Kinase domain
TBK1 (Human)
tbk1 (Danio rerio)

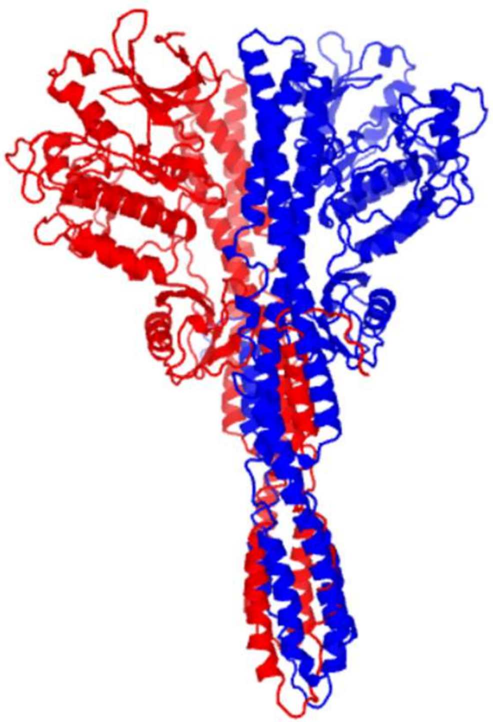

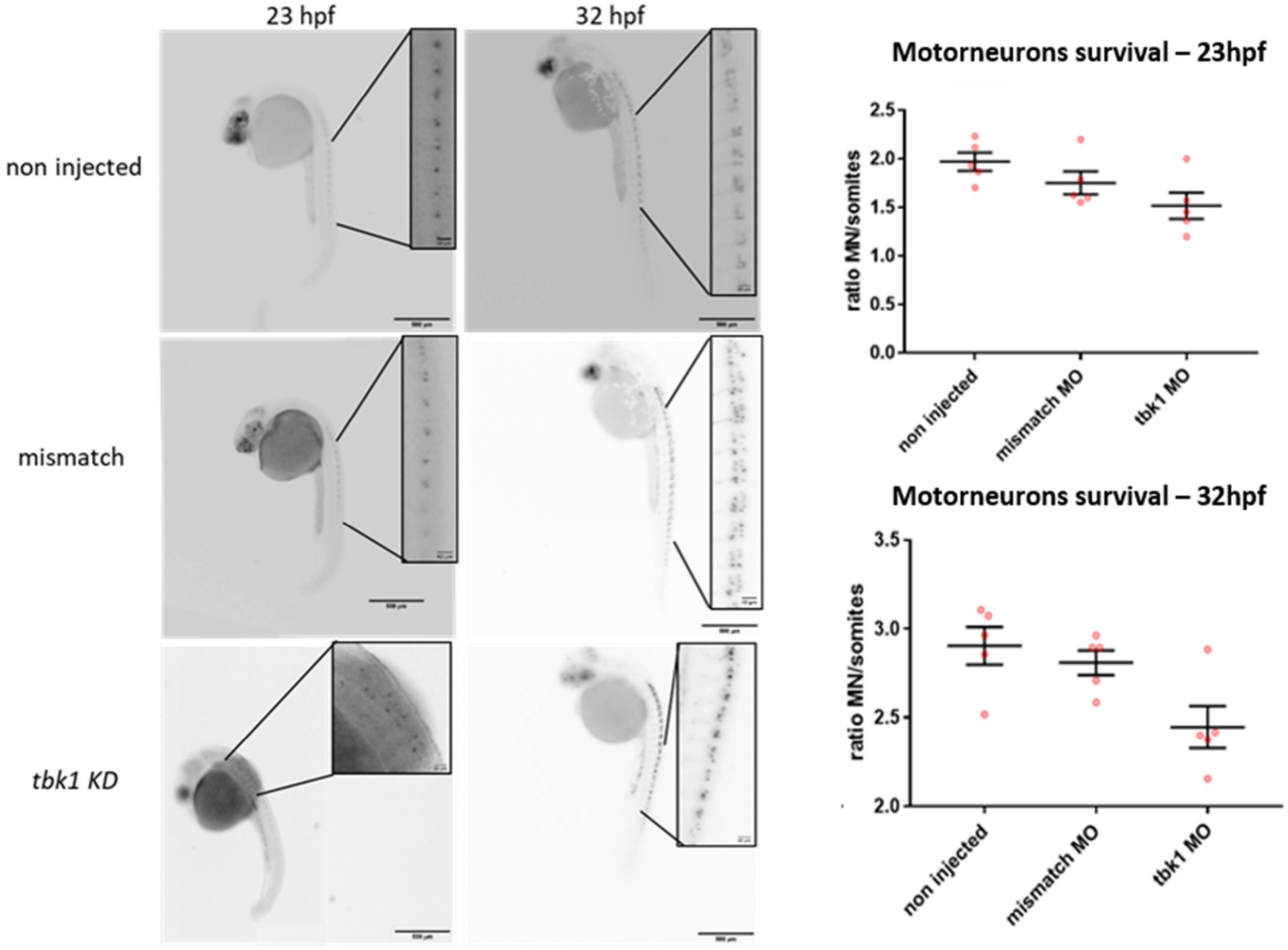

23 hpf
32 hpf
non injected
mismatch
tbk1 KD
Motorneurons survival – 23hpf
ratio MN/somites
2.5
2.0
1.5
1.0
0.5
0.0
non injected
mismatch MO
tbk1 MO
Motorneurons survival – 32hpf
ratio MN/somites
3.5
3.0
2.5
2.0
non injected
mismatch MO
tbk1 MO

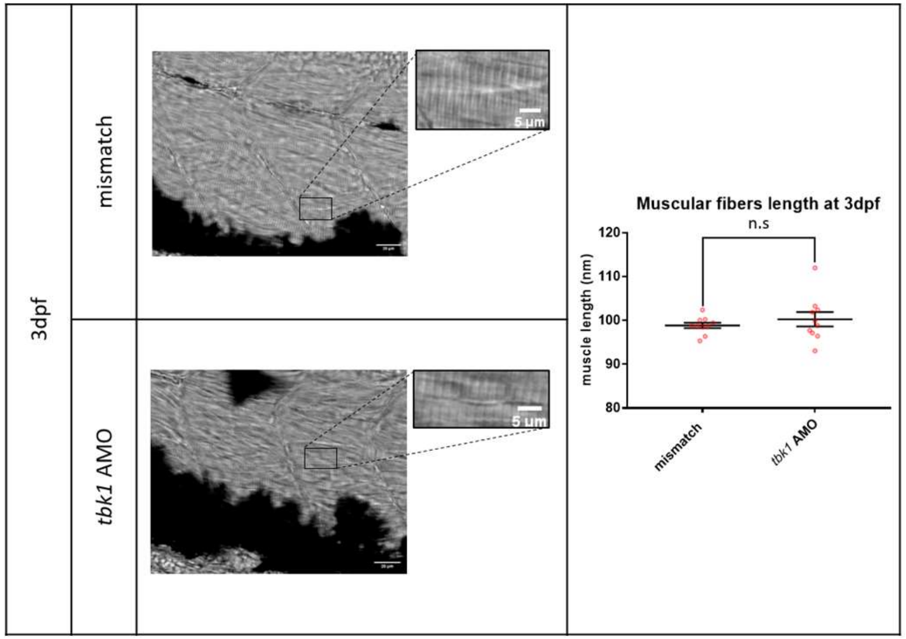
3dpf
mismatch
tbk1 AMO
5 µm
5 µm
Muscular fibers length at 3dpf
n.s
muscle length (nm)
120
110
100
90
80
mismatch
tbk1 AMO

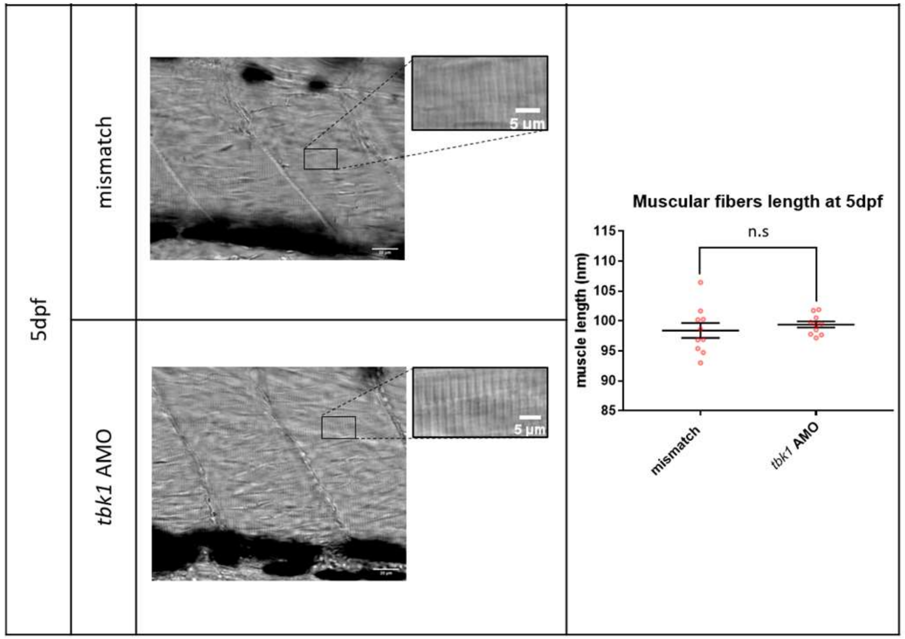
5dpf
mismatch
tbk1 AMO
5 µm
5 µm
Muscular fibers length at 5dpf
n.s
muscle length (nm)
115
110
105
100
95
90
85
mismatch
tbk1 AMO

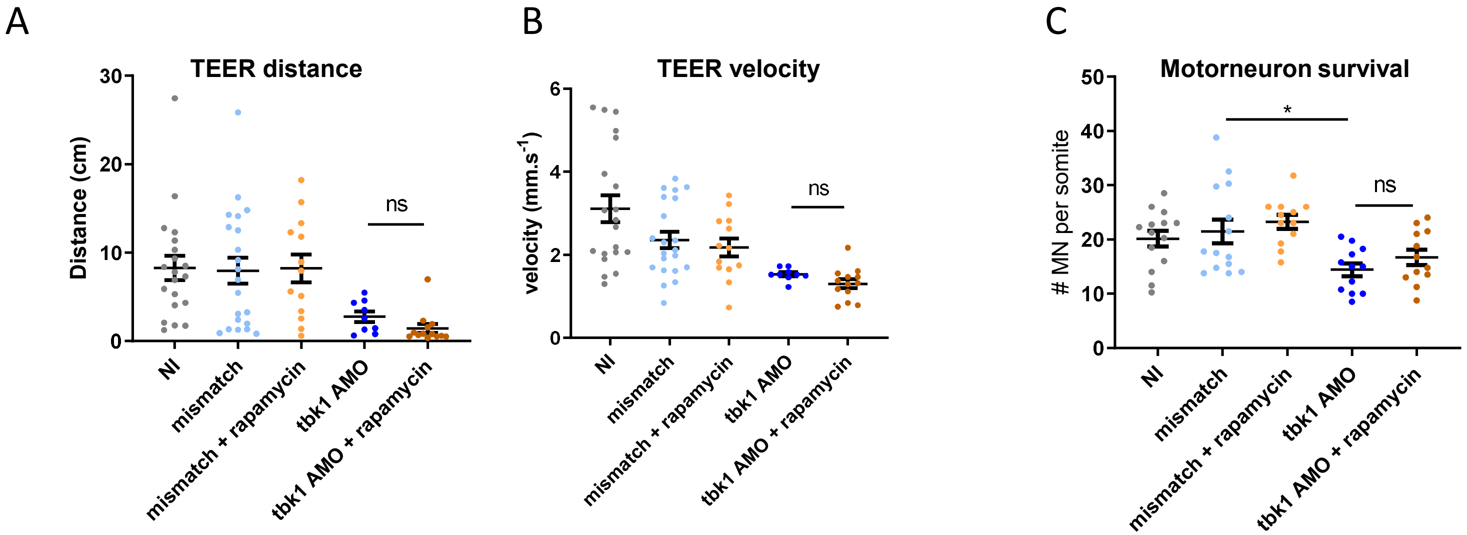
A
TEER distance
Distance (cm)
0
10
20
30
ns
NI
mismatch
mismatch + rapamycin
tbk1 AMO
tbk1 AMO + rapamycin
B
TEER velocity
velocity (mm.s-1)
0
2
4
6
ns
NI
mismatch
mismatch + rapamycin
tbk1 AMO
tbk1 AMO + rapamycin
C
Motorneuron survival
MN per somite
0
10
20
30
40
50
*
ns
NI
mismatch
mismatch + rapamycin
tbk1 AMO
tbk1 AMO + rapamycin

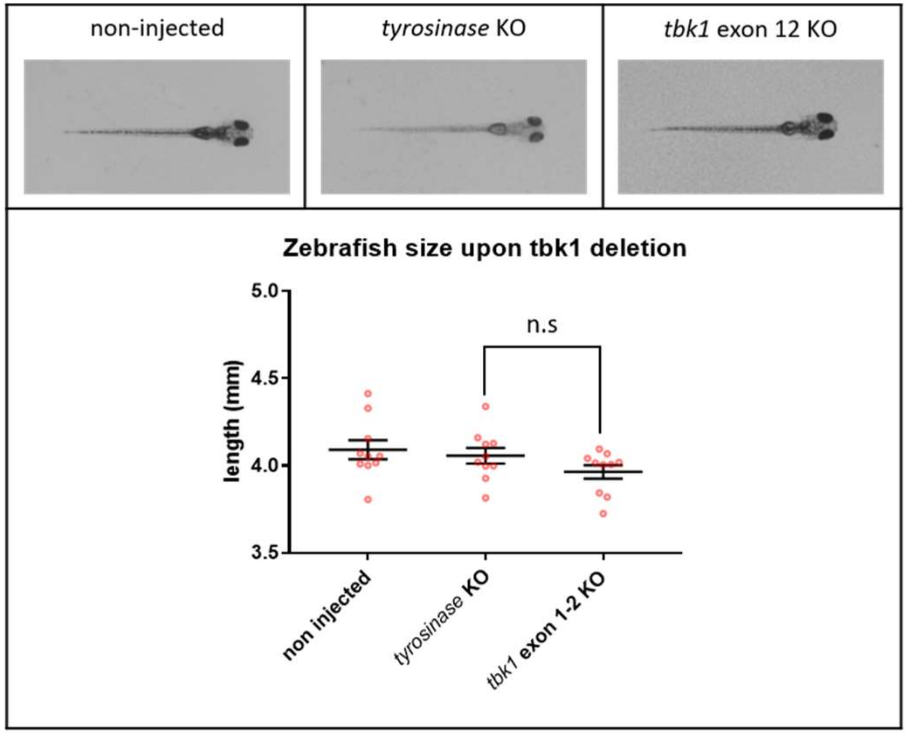

non-injected
tyrosinase KO
tbk1 exon 12 KO
Zebrafish size upon tbk1 deletion
length (mm)
5.0
4.5
4.0
3.5
n.s
non injected
tyrosinase KO
tbk1 exon 1-2 KO

- Supplementary Figure 1. Left panel - TBK1 conservation in zebrafish. Alignment of amino acid sequences of human TBK1 and zebrafish tbk1 orthologs using the PRofile ALIgNEment (PRALINE) multiple sequence alignment application (black line = kinase domain). Right panel - Oligomer structure prediction of zebrafish tbk1 based on the cryo-EM structure resolution of the human TBK1 homodimer (using GalaxyHomomer webtool).

- Supplementary Figure 2. Motor neuron survival in zebrafish embryos injected with mismatch or tbk1 AMO

- Supplementary Figure 3. Morphometric analysis of muscle fibers from zebrafish embryos injected with mismatch or tbk1 AMO at 3 and 5 dpf.

- Supplementary Figure 4. Effect of rapamycin on tbk1 knockdown zebrafish. Quantitative measurement of the TEER distance (A) and velocity (B) of zebrafish embryos injected with mismatch or tbk1 AMO after 24 h of incubation with 0.5 µM rapamycin in the embryo water. C) *In vivo* quantification of spinal motor neuron number in 48 hpf transgenic Tg(mnx1:gal4/UAS:RFP) embryos injected with tbk1 or mismatch AMO after 24 h of incubation with 0.5 µM rapamycin in the embryo water.

- Supplementary Figure 5. General development of tbk1 mutant zebrafish larvae. The absence of pigmentation in tyrosinase mutant zebrafish was used as a functional validation of CRISPR-Cas9 experiments (top panel). Size of tyrosinase mutant, tbk1 mutant or NI control zebrafish larvae at 8 dpf (bottom panel).
